# Initial Outcomes of CardioClick, a Telehealth Program for Preventive Cardiac Care: Observational Study

**DOI:** 10.2196/28246

**Published:** 2021-09-09

**Authors:** Neil M Kalwani, Austin N Johnson, Vijaya Parameswaran, Rajesh Dash, Fatima Rodriguez

**Affiliations:** 1 Division of Cardiovascular Medicine Department of Medicine Stanford University School of Medicine Stanford, CA United States; 2 Cardiovascular Institute Stanford University School of Medicine Stanford, CA United States; 3 Department of Health Policy Stanford University School of Medicine Stanford, CA United States; 4 Stanford University School of Medicine Stanford, CA United States

**Keywords:** telehealth, telemedicine, digital health, eHealth, preventive medicine, prevention, outpatient care, cardiovascular disease, cardiology, South Asian

## Abstract

**Background:**

Telehealth use has increased in specialty clinics, but there is limited evidence on the outcomes of telehealth in primary cardiovascular disease (CVD) prevention.

**Objective:**

The objective of this study was to evaluate the initial outcomes of CardioClick, a telehealth primary CVD prevention program.

**Methods:**

In 2017, the Stanford South Asian Translational Heart Initiative (a preventive cardiology clinic focused on high-risk South Asian patients) introduced CardioClick, which is a clinical pathway replacing in-person follow-up visits with video visits. We assessed patient engagement and changes in CVD risk factors in CardioClick patients and in a historical in-person cohort from the same clinic.

**Results:**

In this study, 118 CardioClick patients and 441 patients who received in-person care were included. CardioClick patients were more likely to complete the clinic’s CVD prevention program (76/118, 64.4% vs 173/441, 39.2%, respectively; *P*<.001) and they did so in lesser time (mean, 250 days vs 307 days, respectively; *P*<.001) than the patients in the historical in-person cohort. Patients who completed the CardioClick program achieved reductions in CVD risk factors, including blood pressure, lipid concentrations, and BMI, which matched or exceeded those observed in the historical in-person cohort.

**Conclusions:**

Telehealth can be used to deliver care effectively in a preventive cardiology clinic setting and may result in increased patient engagement. Further studies on telehealth outcomes are needed to determine the optimal role of virtual care models across diverse preventive medicine clinics.

## Introduction

The COVID-19 pandemic has accelerated the adoption of telehealth and will likely lead to the permanent inclusion of virtual care delivery models in health care systems [1-3]. Cardiovascular disease (CVD) is the leading cause of death in the United States, and as such, it is critical to understand the impact of telehealth on CVD prevention [4]. There are many barriers to in-person care in preventive cardiology clinics, including travel time and the economic costs of time taken off from work for appointments [5,6]. These barriers along with limited engagement among asymptomatic patients with high CVD risk often lead to forgoing of preventive care [7]. Telehealth offers a mechanism to address these barriers [8,9].

Previous studies have suggested that telehealth is as effective as traditional in-office visits in improving the control of chronic diseases such as diabetes [10]. Telehealth adaptation for CVD prevention has been limited but has yielded promising results [11]. Telehealth programs for *secondary* prevention of CVD have achieved equal or superior reductions in risk factors when compared to those achieved in traditional in-person clinics and cardiac rehabilitation [12-14]. Evidence supporting the efficacy of telehealth in *primary* CVD prevention, however, is less conclusive. A randomized controlled trial of a web-based lifestyle intervention for patients with familial hypercholesterolemia did not find significant improvements in CVD risk factors, and a meta-analysis evaluating telehealth in primary CVD prevention concluded that there was insufficient evidence to determine its effectiveness [15,16].

There is some evidence that substituting telehealth visits for in-person visits in preventive cardiology clinics may be effective. An observational evaluation of clinic-based telehealth follow-up visits for CVD risk reduction in remote Saskatchewan, Canada found increased visit completions and similar risk reductions with telehealth as compared to that with usual care, but this study included only 9 patients in the intervention arm [17]. Further research is needed to evaluate telehealth care models in primary CVD prevention. In this study, we describe the early results of CardioClick, a novel telehealth CVD prevention program launched in 2017 in the Stanford South Asian Translational Heart Initiative (SSATHI), a preventive cardiology clinic focused on high-risk South Asian patients based at Stanford Health Care in Northern California.

## Methods

The SSATHI clinic consists of a multidisciplinary care team of cardiologists, an insulin resistance specialist, and registered dieticians. Patients enrolled in the SSATHI prevention program complete initial visits and 2 follow-up visits with a physician and a dietician. Patients undergo a comprehensive risk assessment, including an advanced cardiometabolic panel comprising lipid subfraction, inflammatory markers, lipoprotein(a), and apolipoprotein B/A1 ratio tests. Patients then receive personalized treatment focused on intensive risk reduction through lifestyle interventions and pharmacotherapy, including treatment of hypertension and hyperlipidemia as per the American College of Cardiology and American Heart Association guidelines. Dieticians act as lifestyle intervention specialists in the areas of Nutrition and metabolism, Exercise, Sleep, Transcend stress management (NEST) and medication adherence. A lifestyle questionnaire is administered at the beginning and the end of the program. Based on questionnaire responses, cardiometabolic risk factors, and patient engagement, culturally tailored lifestyle recommendations are provided at each visit. Program completion is defined as completion of baseline and follow-up laboratory tests and at least 2 physician and dietician visits.

In 2017, CardioClick, a novel telehealth clinical pathway, was implemented in SSATHI. Patients enrolled in CardioClick participated in the same prevention program as traditional SSATHI patients, but all follow-up visits with physicians and dieticians were provided as video visits rather than in-person visits (Figure S1 in Multimedia Appendix 1). Patients aged 18-63 years were eligible, limiting enrollees to those with private insurance, as Medicare did not reimburse for video visits at the time. All eligible SSATHI patients were consented for enrollment in CardioClick by default. If they did not wish to enroll, they were offered traditional in-person care. Health care providers were trained to use the video visit platform. Patient access to video visits was enabled through the Stanford Health Care MyHealth mobile app. Patients could complete video visits from a smartphone, tablet, or computer workstation.

The demographic and clinical data for the cohort of CardioClick patients and a historical cohort of patients enrolled in the in-person SSATHI prevention program were manually extracted from the electronic medical records. Patients included in the historical in-person cohort were limited to those aged 18-63 years. Both cohorts included all patients enrolled sequentially between May 2017 and February 2019 for CardioClick and between January 2014 and July 2019 for the historical in-person cohort. Video and in-person physician and dietician follow-up visits were scheduled by the same clinic coordinator at the time of the initial visit or subsequently by phone. All follow-up visits were scheduled for 30-minute slots and were delivered by the same group of physicians and dieticians for both cohorts.

Statistical analyses were completed using SPSS Statistics package (IBM Corp). Unpaired two-tailed *t* tests were used to compare the baseline continuous variables, and chi-square tests were used to compare the categorical variables. Paired two-tailed *t* tests were used to assess within cohort changes in CVD risk factors at follow-up, and unpaired two-tailed *t* tests were used to compare changes between cohorts. *P* values less than .05 were deemed statistically significant. This study was approved by the Stanford Institutional Review Board.

## Results

The CardioClick cohort consisted of 118 patients and the historical in-person cohort consisted of 441 patients. CardioClick patients were older (43 years vs 41 years, respectively; *P*=.009) and had lower baseline triglyceride levels (113 mg/dL vs 134 mg/dL, respectively; *P*=.01) than the in-person cohort patients. The cohorts were otherwise similar in terms of demographics, comorbidities, and baseline CVD risk factors (Table 1).

**Table 1:** Baseline characteristics and completion rates of the preventive cardiac care program.

Characteristics	CardioClick cohort (n=118)	Historical in-person cohort (n=441)	*P* value^a^
Age (years), mean (SD)	43 (9)	41 (9)	.009
Gender (male), n (%)	94 (79.7)	375 (85.0)	.66
Diabetes, n (%)	9 (7.6)	43 (9.8)	.48
Hypertension, n (%)	31 (26.3)	101 (22.9)	.47
Smoker, n (%)	24 (20.3)	93 (21.1)	.86
Systolic blood pressure (mm Hg), mean (SD)	124 (13)	124 (15)	.79
Diastolic blood pressure (mm Hg), mean (SD)	78 (10)	80 (9)	.30
Total cholesterol (mg/dL), mean (SD)	185 (43)	193 (46)	.09
Low-density lipoprotein cholesterol (mg/dL), mean (SD)^b^	119 (40)	124 (40)	.18
High-density lipoprotein cholesterol (mg/dL), mean (SD)	48 (14)	48 (13)	.84
Triglycerides (mg/dL), mean (SD)	113 (61)	134 (78)	.01
Hemoglobin A_1c_ (%), mean (SD)^b^	5.7 (0.7)	5.7 (0.9)	.61
BMI (kg/m^2^), mean (SD)	27 (4)	27 (4)	.27
Completed program, n (%)	76 (64.4)	173 (39.2)	<.001

^a^*P* values are for between-group comparisons.

^b^Reported for only those patients for whom data were available.

**Figure 1:**
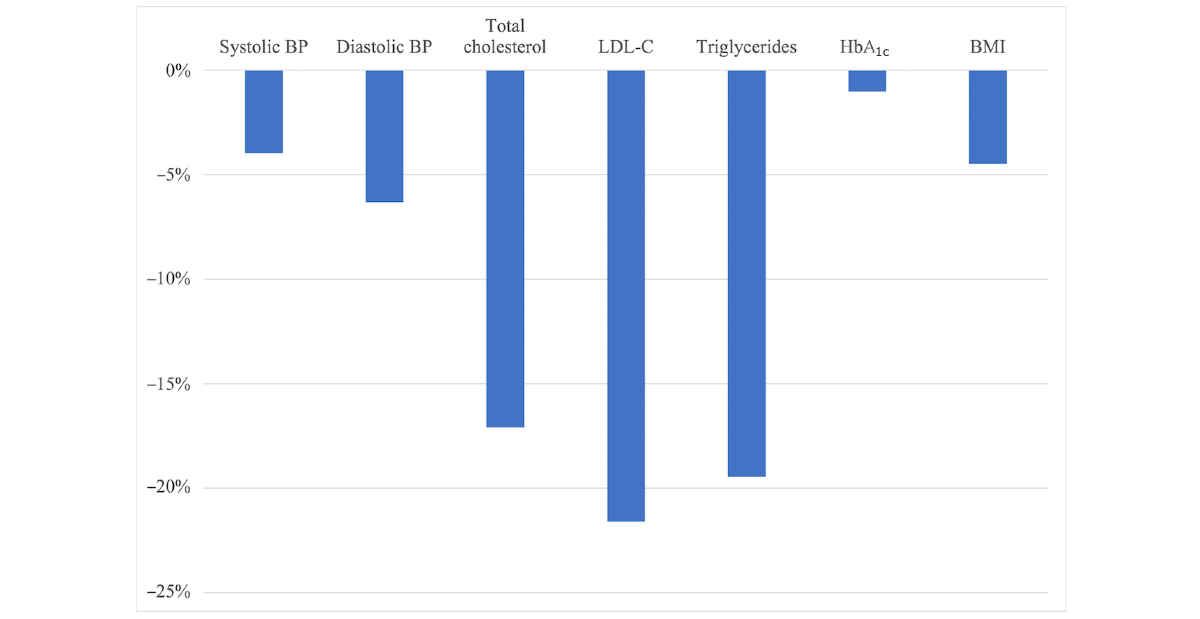
Percentage change in the cardiovascular disease risk factors in CardioClick patients at the completion of the preventive cardiac care program. The changes in all the cardiovascular disease risk factors, except HbA_1c_, were statistically significant at *P*<.05. BP: blood pressure; HbA_1c_: hemoglobin A_1c_; LDL-C: low-density lipoprotein cholesterol.

With the introduction of CardioClick, patients were significantly more likely to complete the clinic’s prevention program (76/118, 64.4% vs 173/441, 39.2%, respectively; *P*<.001) and they did so in lesser time (mean 250 days vs 307 days, respectively; *P*<.001) than the in-person cohort patients. CardioClick patients were also more likely to utilize clinic services. Of the 3 dietician visits offered, the typical CardioClick patient completed all 3 visits, while the typical patient in the historical in-person cohort only completed 1 visit. Patients who completed CardioClick experienced significant reductions in CVD risk factors, including systolic and diastolic blood pressure, total cholesterol levels, low-density lipoprotein cholesterol levels, triglyceride levels, and BMI (*P*<.001 for all) (Figure 1). In contrast, there was no reduction in BMI observed among patients who completed the prevention program in the historical in-person cohort, and the observed reduction in total cholesterol levels for these patients was lower than that observed in CardioClick patients (–19 mg/dL vs –33 mg/dL, respectively; *P*=.02). Reductions in other risk factors were not significantly different between patients completing the prevention program in the 2 cohorts (Table 2).

**Table 2:** Changes in the cardiovascular disease risk factors at the completion of the preventive cardiac care program.

Risk factors	CardioClick cohort (n=76)					Historical in-person cohort (n=173)					Comparison of change between cohorts, *P* value
	Baseline	Follow-up	Change	Within cohort, *P* value	Baseline		Follow-up	Change	Within cohort, *P* value		
Systolic blood pressure (mm Hg), mean (SD)	126 (17)	121 (13)	–5 (11)	<.001	128 (15)		125 (13)	–3 (13)	.005	.24	
Diastolic blood pressure (mm Hg), mean (SD)	79 (10)	74 (9)	–5 (11)	<.001	81 (10)		79 (8)	–2 (10)	.009	.05	
Total cholesterol (mg/dL), mean (SD)	193 (41)	160 (38)	–33 (45)	<.001	190 (47)		171 (43)	–19 (44)	<.001	.02	
Low-density lipoprotein cholesterol (mg/dL), mean (SD)^a^	125 (36)	98 (33)	–27 (40)	<.001	121 (39)		104 (37)	–17 (38)	<.001	.06	
High-density lipoprotein cholesterol (mg/dL), mean (SD)	50 (14)	49 (11)	–1 (8)	.22	48 (14)		49 (14)	0.8 (7)	.11	.08	
Triglycerides (mg/dL), mean (SD)	113 (55)	91 (40)	–22 (44)	<.001	135 (83)		112 (67)	–23 (82)	<.001	.92	
Hemoglobin A_1c_ (%), mean (SD)^a^	5.8 (0.6)	5.8 (0.5)	–0.1 (0.5)	.52	6.0 (1.0)		5.9 (0.1)	–0.1 (0.5)	.23	.86	
BMI (kg/m^2^), mean (SD)	26.8 (4)	25.6 (3)	–1.2 (1.2)	<.001	26.7 (3.7)		26.7 (4.7)	0.01 (3.5)	.96	.004	

^a^Reported only for those patients for whom data were available.

## Discussion

### Principal Findings

The use of telehealth has rapidly expanded as a result of the COVID-19 pandemic; however, there has been limited and inconclusive evidence on the effectiveness of telehealth in primary CVD prevention [11,15]. The initial results of CardioClick presented here precede the COVID-19 pandemic and suggest that telehealth is an effective way for delivering preventive cardiac care and may enhance patient engagement. Following implementation, CardioClick became the default care pathway in the SSATHI clinic. We found that CardioClick patients were similar demographically and clinically to a historical cohort of SSATHI patients who received in-person care, suggesting against significant selection bias in enrollment. Importantly, patients in both cohorts were treated by the same group of health care providers using the same prevention program framework.

CardioClick patients completed the prevention program at a higher rate and in a shorter amount of time on average, suggesting that telehealth may decrease barriers to care in settings with readily available in-person specialty care and not just in low-access settings, as has been demonstrated previously [18]. We found that CardioClick participants who completed the prevention program achieved reductions in CVD risk factors, including blood pressure, lipid concentrations, and BMI, which matched or exceeded the reductions observed in the historical in-person cohort. Notably, the reductions in blood pressure achieved exceed those previously reported in the literature for several telehealth primary prevention lifestyle interventions [11,15,16].

To our knowledge, this study represents the first evaluation of a telehealth care model for primary CVD prevention in a clinic setting with patients completing visits from home. The risk factor reductions observed support the continued use of telehealth in preventive medicine clinics and motivate further study of its impact on care delivery and outcomes.

### Limitations

This study should be interpreted in the context of several limitations. The intervention was implemented at a single center with a patient population that was majority male, middle-aged, and of South Asian origin. It is unclear whether the observed reductions in CVD risk factors would be generalizable to diverse patient populations. Although patients were enrolled in CardioClick by default, there may exist unobserved differences between the cohorts, which accounted for the observed differences in the outcomes. Finally, it is unknown whether the risk factor reductions achieved will be sustained in long-term follow-up.

### Conclusions

Implementation of CardioClick, a clinic-based telehealth primary CVD prevention program, was associated with increased patient engagement and significant reductions in risk factors among those completing the program. The success of this initiative suggests that telehealth can be utilized to deliver care effectively in the preventive cardiology clinic setting. Further research is needed assessing telehealth outcomes with randomized evaluations across diverse patient populations.

## Appendix

Multimedia Appendix 1 CardioClick prevention program overview.

## Abbreviations

CVD: cardiovascular disease
NEST: Nutrition and metabolism, Exercise, Sleep, Transcend stress management
SSATHI: Stanford South Asian Translational Heart Initiative
