# Supplementary figures and images for: Initial Outcomes of CardioClick, a Telehealth Program for Preventive Cardiac Care: Observational Study
Source: JMIR Cardio. 2021 Sep 9;5(2):e28246. doi: 10.2196/28246 (PMC8461530; doi:10.2196/28246)

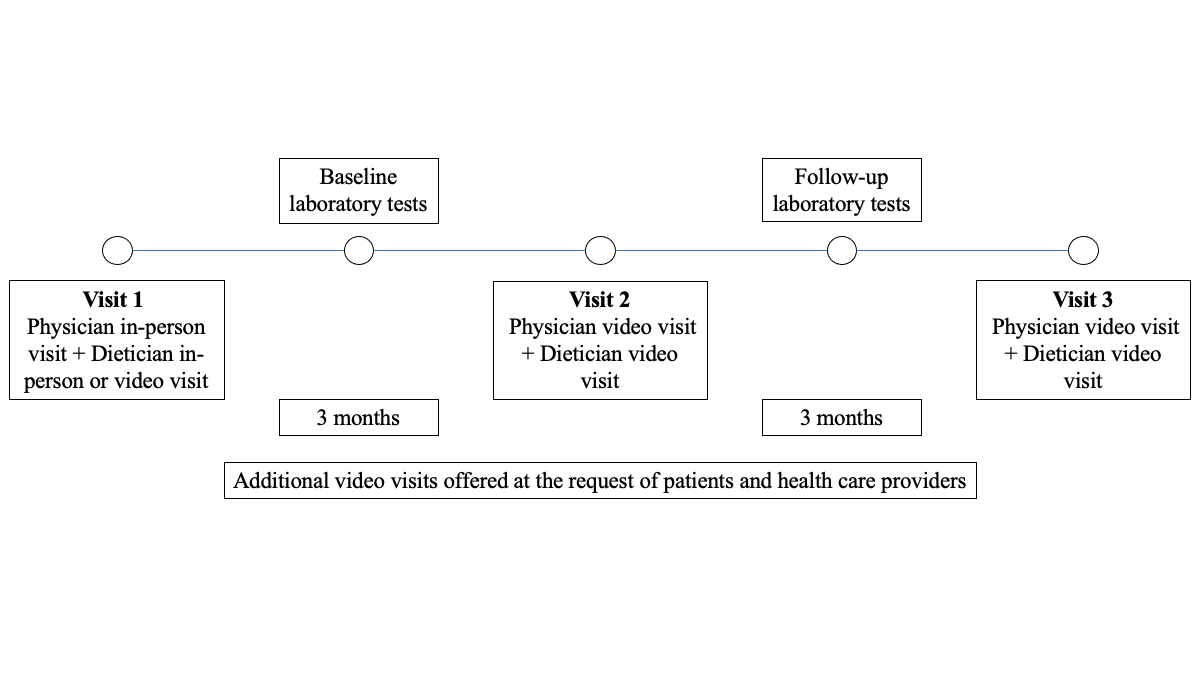

Supplement: Multimedia Appendix 1 [file cardio_v5i2e28246_app1.png]
